# Collective Action for Wellness in the Malaysian Workplace: Protocol for a Feasibility Study

**DOI:** 10.2196/39238

**Published:** 2022-12-05

**Authors:** Janus Y Thai, Tracy McCaffrey, Amutha Ramadas, Dharshani Chandrasekara, Sharon G M Koh, Tammie Suet Ting Choi, Hema Malini, Jue Xie, Patrick Olivier, Anuar Zaini Md Zain, Jessica Watterson

**Affiliations:** 1 Jeffrey Cheah School of Medicine and Health Sciences Monash University Malaysia Subang Jaya Malaysia; 2 Department of Nutrition, Dietetics and Food Monash University Melbourne Australia; 3 Department of Human-Centred Computing Monash University Melbourne Australia; 4 Department of Economics Monash University Malaysia Subang Jaya Malaysia; 5 Faculty of Nursing Andalas University Padang City Indonesia

**Keywords:** workplace wellness, healthy behaviours, chronic diseases, digital health, Malaysia, wellness, workplace, disease, digital wellness, digital, promote, health knowledge, diet, employee, mobile device, intervention, social, social group

## Abstract

**Background:**

Chronic diseases and the associated risk factors are preventable with lifestyle changes such as eating a healthier diet and being more physically active. In Malaysia, the prevalence of chronic diseases, including diabetes, hypertension, and heart diseases, has risen. In the present study, we explore the potential of co-designing and implementing a digital wellness intervention to promote socially-driven health knowledge and practices in the workplace in Malaysia, drawing on social cognitive theory, social impact theory, and social influence theory.

**Objective:**

This study aims to co-design and assess the feasibility of a socially-driven digital health intervention to promote healthy behavior and prevent chronic diseases in a workplace in Malaysia.

**Methods:**

This study involves two phases: (i) identifying the barriers and facilitators to healthy behaviors at work and co-designing the intervention activities with the employees, (ii) implementing and evaluating the intervention’s feasibility. Phase 1 will involve qualitative data collection and analysis through semi-structured, in-depth interviews and co-design workshops with the employees, while Phase 2 will consist of a feasibility study employing quantitative measurements of health behaviors through accelerometers and questionnaires.

**Results:**

This study was funded in June 2021 and ethics approval for Phase 1 was obtained from the Monash University Human Research Ethics Committee in January 2022. As of August 2022, qualitative interviews with 12 employees have been completed and the data has been transcribed and analyzed. These results will be published in a future paper with results from all Phase 1 activities.

**Conclusions:**

The study will help us to better understand the mechanisms through which digital technologies can promote socially-driven health knowledge and behaviors. This research will also result in a scalable wellness intervention that could be further tailored and expanded to other employers and social groups across the region.

**International Registered Report Identifier (IRRID):**

PRR1-10.2196/39238

## Introduction

### Background

Chronic diseases, such as diabetes, hypertension, and cardiovascular disease, have been a growing public health concern in the Southeast Asian region [1]. In Malaysia, the prevalence of diabetes in adults rose to 18.3% in 2019. About 8% of the adult population in Malaysia, or 1.7 million people, have risk factors for diabetes, hypertension, and hypercholesterolemia [2,3]. These chronic diseases and associated risk factors are preventable or manageable with lifestyle changes, such as eating a healthier diet, getting more exercise, quitting smoking, and reducing stress.

Previous studies suggest that workplace wellness initiatives can benefit individuals and employers through lower health care costs and reductions in absenteeism [4-6]. As the working age population is large and many adults spend the majority of their daytime hours at work, the workplace has been recognized as a favorable setting for health promotion. The World Health Organization has also identified workplace wellness programs as a strategic method to prevent and treat noncommunicable diseases (NCDs) [7]. Further evidence suggests that peer support, such as that found from friends, family, or colleagues, can be more effective at producing behavior change [8]. Messages with health information or peer support can also serve as a “nudge” to influence healthier daily choices [9].

From activity trackers that monitor physical activity levels to smartphone apps that provide personalized health education, the incorporation of digital technologies is becoming commonplace, especially in this digital age. Digital health interventions have been shown to have a positive effect on workplace health promotion [10-12]. Over the past 2 decades, growing research has been conducted to evaluate the effectiveness and feasibility of digital health interventions in the workplace context of many countries [13-15], but relatively few studies have been conducted in low- and middle-income countries (LMICs). Among the broad range of health issues that can be addressed by digital workplace wellness tools, NCDs are one of the most common areas of focus, likely due to the fact that behaviors such as eating and sitting are common in workplaces and contribute to NCD risk. Building on this body of research, this study will use digital tools in the workplace to facilitate health promotion and leverage the fact that 91% of people in Malaysia have smartphones and use messaging apps like WhatsApp and Facebook Messenger regularly [16,17].

However, the global coronavirus SARS-CoV-2 (COVID-19) pandemic has created unprecedented challenges to the research activities of this study. To stem the rising number of COVID-19 cases, countries around the world have announced nationwide quarantines and put people on various forms of lockdown. In Malaysia, movement control orders have been imposed by the Malaysian government from March 18, 2020, with a series of restrictions on movement and mass assembly. As of February 17, 2022, there were a total of 3,111,514 confirmed cases of COVID-19 with 32,201 deaths in Malaysia reported to the World Health Organization [18]. Owing to movement restrictions and frequent changes from COVID-19, we have developed alternative plans for all study activities, including interviews, co-design workshops, and intervention testing to be carried out remotely, if needed.

### Aims

Our project aims to develop and assess the feasibility of a digital intervention (referred to for now as Collective Action for Wellness in the Workplace [CAWW]) to improve employees’ health behaviors and well-being, thus reducing the risk of chronic diseases in the workplace. Based on the current evidence, there is a need to develop sustainable interventions to support employees’ well-being in LMICs. In the current project, we will focus on co-designing and implementing an intervention in a Malaysian company, drawing on social cognitive theory, social impact theory, and social influence theory.

This study has 3 aims. Aim 1 is to understand the barriers and facilitators to healthy behaviors among employees from the target company, both within the workplace and at the work-home interface. Aim 2 is to co-design intervention activities with the employees that deliver collective education and action around healthy behaviors using digital communication or coordination, such as WhatsApp. Aim 3 is to assess the feasibility of measuring health, behavioral, and social outcomes, as well as the cost–benefit to the target company before and after the implementation of the intervention.

### Objectives

In order to obtain the aims outlined above, the study will achieve the following objectives:

- identify factors that influence employees to engage in healthier or less healthy behaviors at work, including social influences from others;
- explore workplace wellness activities and digital communication or coordination tools that are of interest to employees;
- co-design specific wellness activities with employees as part of the CAWW intervention;
- test the implementation of these activities with employees over the course of 6 months; evaluate the feasibility of measuring the outcomes of interest through employees completing questionnaires and wearing fitness trackers; and
- assess the feasibility and acceptability of collecting employee attendance and participation data.

### Research Questions

The following research questions will be examined throughout the study. The qualitative interviews and workshops (phase 1) will investigate the following:

1. What factors influence employees to engage in healthier or less healthy behaviors at work?
2. How do the behaviors, opinions, or actions of others in the workplace influence employees’ health?
3. What types of workplace wellness activities are interesting to employees, and can they be supported or sustained by the organization?
4. Do employees think that digital communication or coordination tools, such as WhatsApp, can be used to coordinate healthier behaviors or activities in the workplace?
5. How can existing social influence within the workplace be leveraged to create new wellness activities and encourage employee participation?

The quantitative feasibility portion of the study (phase 2) will investigate the following:

1. Will employees agree to participate in wellness activities at work, complete questionnaires, and wear fitness trackers, and what proportion will complete these tasks at both baseline and follow-up?
2. Are the selected measures sufficiently sensitive and suitable for measuring the outcomes of interest?
3. What is the variability observed in the outcome measures of interest?
4. Is it feasible to collect data on work attendance from an employer for consenting employees?

## Methods

### Study Setting

This study will be conducted with employees from a Malaysian biotechnology company. The company employs around 2000 people across functions such as research and development, manufacturing, and marketing. Employees from a range of departments and demographic backgrounds will be included in this study. This company provides an interesting setting for this study as its employees reflect the unique multiethnic composition of Malaysia, and includes employees working in desk or laboratory jobs, as well as employees working in manufacturing positions that require more manual labor.

### Study Design

**Figure 1:**
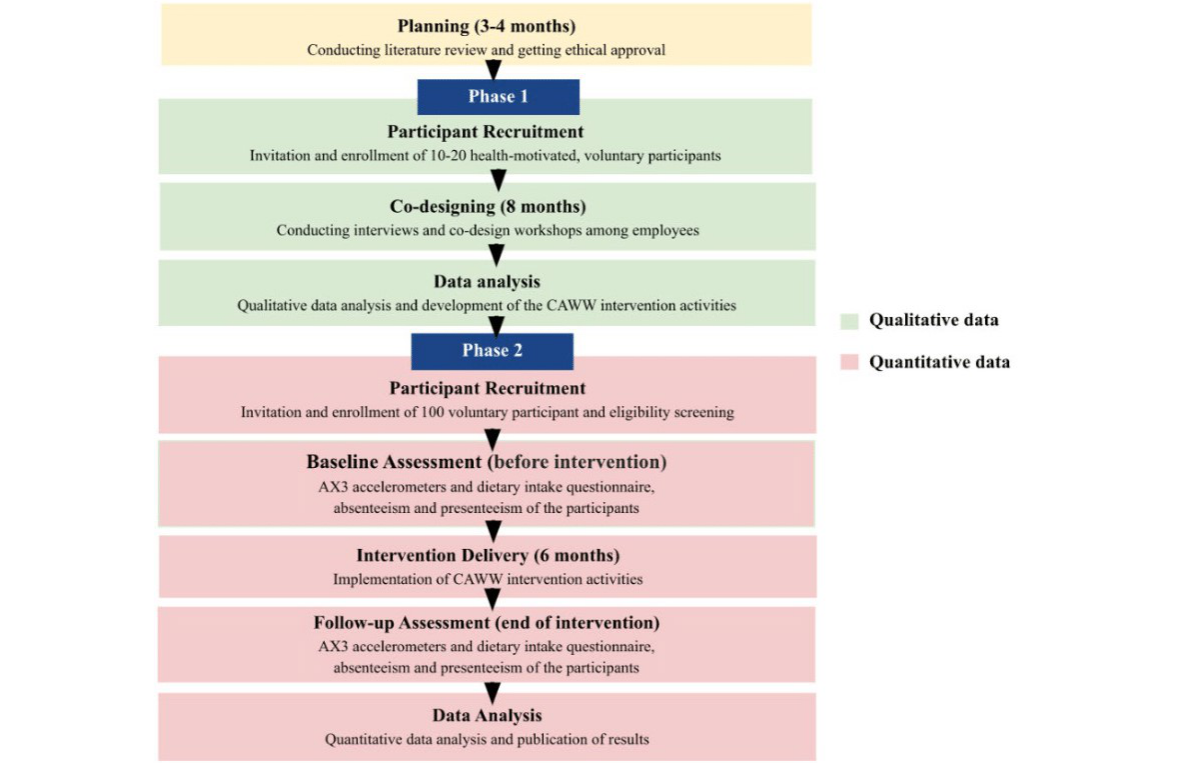
Overview of the timeline of the study. CAWW: Collective Action for Wellness in the Workplace.

This study will employ a mixed method approach and consist of two phases. Phase 1 will consist of 8 months of co-design activities, and phase 2 will consist of a 6-month feasibility study of the CAWW intervention. Figure 1 shows an overview of the timeline of the study.

### Theoretical Framework

The design of the phase 1 interviews, the phase 2 intervention, and our planned phase 2 measurement framework will be informed by the social cognitive theory (SCT) [19], social influence theory [20], and social impact theory [21], which acknowledge that behavior is influenced by factors at both the individual and social levels. Even though most of the past studies of workplace health behavior change in LMICs have focused on individual-level factors [10,22-24], we argue that social-level factors and the environmental context are also equally important in behavior change. SCT was selected as an appropriate theoretical lens for the study owing to its incorporation of individual environmental and behavioral factors. SCT proposes that health behaviors are influenced by dynamic and reciprocal interactions among individual, environmental, and behavioral factors [19]. Individual factors include cognitive, affective, and biological processes, such as knowledge, expectations, learned behaviors, beliefs, and attitudes [25]. Self-efficacy, a key construct of the theory, refers to an individual’s belief in his or her ability to execute a particular behavior necessary to obtain the intended results. Environmental factors include the external social context, which can contribute to observational learning and reinforcements to continue or discontinue a behavior. Finally, behavioral factors, or responses to stimuli to achieve goals, include factors like expectations and capability.

**Figure 2:**
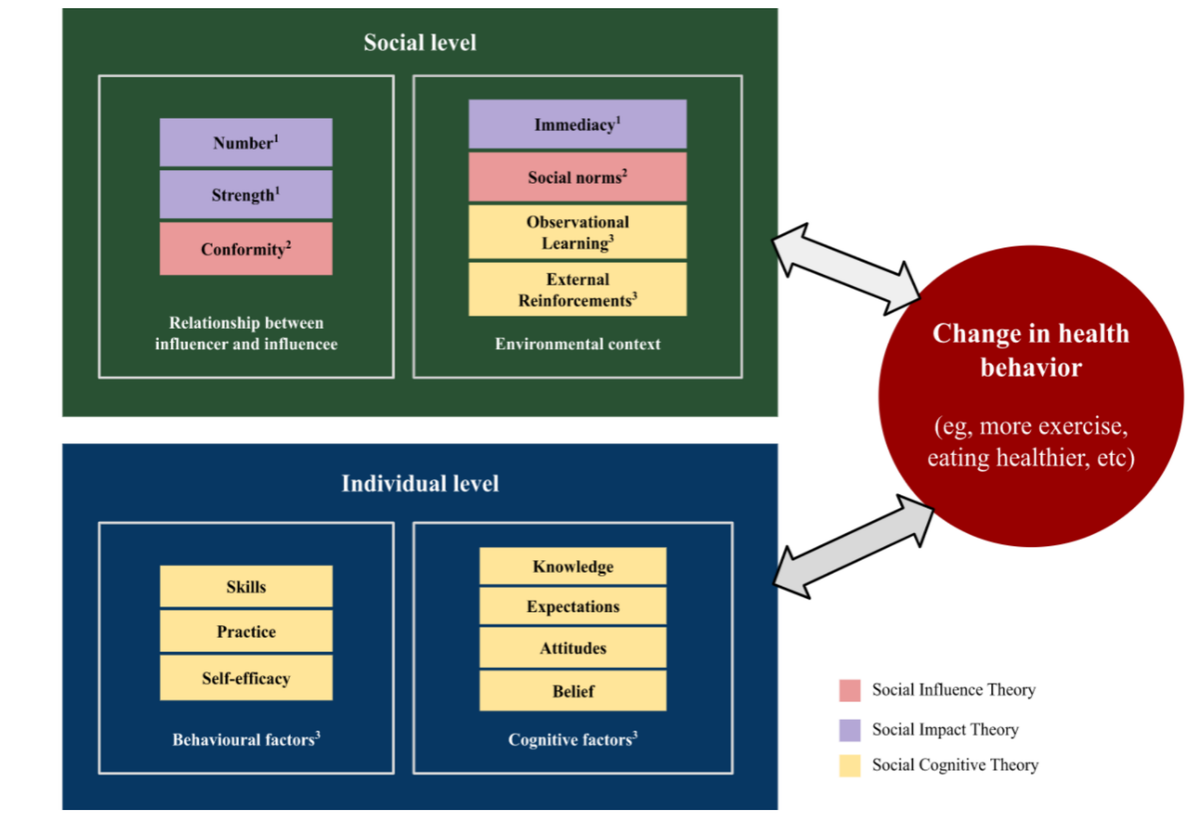
Theoretical constructs influencing changes in health behaviors at the individual and social levels. Sources: ^1^Latané, 1981[21], ^2^Cialdini & Goldstein, 2004 [20], ^3^Bandura, 2011[25].

Our understanding of social-level factors can be further expanded by drawing on the social influence theory, which explains how motivations—to be accurate, to affiliate, and to maintain a positive self-concept—can drive behavior [20]. More specifically, factors such as authority and conformity can influence an individual’s decision to adopt certain behaviors [26,27]. The social impact theory adds that the amount of influence a person experiences in a social context will depend on the strength or power of the group, the psychological or physical proximity of the group (immediacy), and the number of people exerting the influence in the group [21]. These theoretical constructs have been synthesized into a theoretical framework informing this study in Figure 2.

### Phase 1: CAWW Intervention Development using a Qualitative Co-design Approach

Co-design is a process involving the collective creativity of all stakeholders, including researchers, designers, developers, and users [28]. It aims for better design through a rich and deep understanding of the user’s knowledge, behaviors, and thoughts [29]. By employing the co-design methodology, we will engage the company employees from the beginning of the design process, discussing ideas and developing the CAWW intervention activities to be culturally relevant (both at a societal level and fitting with company culture) and meet their true needs [30]. More detail on the co-design methods that will be used is provided in the following sections.

#### Qualitative Interviews

First, qualitative, in-depth, individual interviews will be conducted with a purposive sample of highly motivated and health-focused employees (target N=10-15). The sample will be balanced by age, gender, ethnic groups, and working departments. The purposive sampling of motivated and health-focused individuals is based on the idea that these employees may be more likely to help identify new ideas and opportunities for a healthier workplace. A few pilot interviews will be conducted prior to the actual interview to refine the interview questions. The research team members or a trained research assistant will administer the interviews and follow a semistructured protocol that will be developed from primary research questions, the theoretical framework, and project goals. In relation to the theoretical framework, questions about how individual-level and social-level factors shape employees’ perceptions and workplace health behaviors were included in the interview protocol. For example, to explore the social-level relationships between influencers and influencees, participants will be asked, “Who are the key people that make decisions or influence people in your workplace? Why are they influential?” Each interview will take approximately 60 minutes and will be conducted via an web-based platform (eg, Zoom; Zoom Video Communication Inc) without the involvement of a third party to ensure confidentiality. The interview will be conducted in English or Malay based on the participants’ language preferences and transcribed using Descript software. All data will be stored in an institution-approved secure space.

#### Co-design Workshops

Second, we will conduct 2 co-design workshops with employees to design the format and content of the intervention. A target of 10-15 participants will be recruited to participate in each co-design workshop. Flyers will be sent by the human resources (HR) team to recruit voluntary and interested employees. Depending on the current unstable pandemic context of COVID-19 in Malaysia, the co-design workshops will be held through either the Zoom platform or face to face in a private room of the company.

The activities of the workshops will be governed by the findings from the qualitative interviews. For instance, based on the interview data, we will identify which variables of the theoretical framework are more prominent in encouraging individuals to practice healthy behaviors in the workplace. Following this, the workshop activities will be designed to incorporate these variables. The participants of the co-design workshops will then be asked to help design workplace wellness activities integrating the variables that encourage the employees to focus more on their health.

The first workshop will be held with the company’s HR department to identify the nature of the activities that can be sustainably supported and financed. Initial ideas for evidence-based intervention activities will be shared with the group, based on a review of the literature on workplace wellness interventions. The employees from the HR department were selected for the first workshop as it is likely that they will be able to provide more holistic input from their perspectives both as employees and HR professionals, owing to their dual role in the company. For the second workshop, we will conduct an activity with other company employees outside the HR department to refine and prioritize potential activities resulting from the first workshop.

#### Phase 1 Data Analysis

Following transcription of the interview data, a reflexive thematic analysis approach [31] will be used. In reflexive thematic analysis, the researchers deeply immerse themselves in the collected data and follow an “organic” process through “reflexive interpretation” [32]. Given that reflexive thematic analysis encourages researchers to actively engage with research data and make transparent decisions, all decisions from data collection to data analysis in phase 1 will be designed to align with strategies outlined in this approach. For example, when determining the sample size, we will pay attention to the adequacy of the data gathered from interviews in relation to the purpose of our research, the relevance of the gathered data to research questions, and whether the sample is representative of different departments within the organization [32]. Thus, the target sample size (N=10-15), which has currently been determined based on the representation of workers from different departments, may be modified as necessary during data collection depending on the richness and complexity of data gathered from the interviews in relation to the research questions. Further, since the study is informed by a theoretical framework designed by considering individual, cognitive, social, and environmental factors that influence health behavior change, we will adopt a theoretical (deductive) approach in conducting the reflexive thematic analysis [33].

Employee input during the workshops will be recorded and subsequently analyzed as well. Common themes will be concluded from the findings across workshops, and the final components of the CAWW intervention will be identified via consensus. With the input from the participants during the co-design phase, the research team will identify the intervention wellness activities, the potential channels (eg WhatsApp, Zoom, in-person, etc) to coordinate and conduct the wellness activities, ways to motivate participation in wellness activities, and the timing and frequency of the wellness activities.

### Phase 2: CAWW Testing and Evaluation of Acceptability and Feasibility Using a Quantitative Approach

Phase 2 will consist of testing the feasibility of the intervention through baseline data collection, followed by the 6-month intervention, and then the collection of follow-up data. Participation data (number of messages sent, attendance to events, etc) will be collected throughout the intervention. In this phase, we aim to enroll 100 participants in the feasibility study. The study was funded to include 100 participants. This is a conservative sample size for a feasibility study and is comparable to many other feasibility and pilot studies, given the lack of consensus on the best methods to calculate sample size for a feasibility study [34]. Employees will be invited by their employer to contact the research team if they are interested in participating. Participants with the following criteria will be eligible for inclusion in the study: adults aged 18 years and older, are employed through the study completion date, do not have a mobility impairment, working in the office or at home at least 3 days a week, and have access to a mobile device. Participants who are away on extended leave for more than 2 weeks or who are pregnant or lactating will be excluded from this study. Additional employees can participate in the intervention activities, but their data will not be collected. Informed consent will be obtained from participants who meet the inclusion criteria and participation will be voluntary. Financial incentives will be provided for participation in research activities including surveys and data collection, but no compensation will be given for participating in the intervention wellness activities.

#### Study Measures

The research team will collect data at baseline before the intervention starts and at follow-up after the intervention ends (after 6 months). The baseline and follow-up data collection will include 4 aspects: (1) health, including health-related quality of life (HRQoL); (2) behaviors, including physical activity and dietary intake; (3) social, including employees’ sense of community [35]; and (4) economic, including absenteeism, presenteeism, and total cost of implementing the program for employers.

#### Primary Outcomes

The primary measured outcomes of this study are physical activity and diet. The AX3 accelerometer will be used on wristbands to monitor the physical activity levels of participants for a 7-day period at baseline and follow-up. Participants’ dietary data will be collected at baseline and follow-up through a self-administered questionnaire that asks about participants’ food and beverage consumption on the previous day. This questionnaire, the Diet Quality Questionnaire, has been developed and validated to measure diets worldwide as part of the Global Diet Quality Project, and the country-specific questionnaire for Malaysia will be used [36].

#### Secondary Outcomes

The HRQoL will be measured through an extensively used questionnaire, the Short Form-12 Health Survey Version 2 (SF-12v2). The SF-12v2 was developed from the Medical Outcomes Study (MOS) 36-item Short-Form Health Survey (SF-36) [37], and its brevity reduces the burden on participants and researchers [38]. The 12-item SF-12v2 has also been shown to be reliable and valid in measuring HRQoL of a wide range of population groups [38-42]. The employees’ sense of community will be assessed using Sense of Community Index version 2 (SCI-2) [43]. SCI has been frequently used as a quantitative measure of a sense of community, being proven as a strong predictor of behaviors and a valid measurement tool. It has been used with different cultures in many different contexts [43]. The employees’ health-related productivity will be assessed through absenteeism and presenteeism. Absenteeism will be examined through recorded sick days taken in the previous 6 months (reported by the employer at baseline and follow-up). Sickness absence by definition is the absence from work owing to illness and has been used as the measure of health and well-being in the population [44-46]. Presenteeism will be measured using the 6-item Stanford Presenteeism Scale (SPS-6) [47]. SPS-6 has strong psychometric characteristics in measuring health and productivity [47]. Finally, data will also be collected on the estimated costs of the intervention.

#### Feasibility and Acceptability

Satisfaction with the intervention will be assessed by a modified version of the client satisfaction questionnaire (CSQ) [48]. The 8-item CSQ has been recognized as a useful measure of general satisfaction and takes only 3-8 minutes to complete [48]. It is associated with therapists’ estimates of client satisfaction and has good reliability due to its high internal consistency. The CSQ will be modified to fit the workplace context and to measure the general satisfaction with the intervention activities.

#### Phase 2 Data Analysis

For the quantitative outcome measures in phase 2, a descriptive analysis will be conducted. As this is only a feasibility study and the sample size is appropriately small, we will not make statistical inferences on the effectiveness of the intervention [49]. The data will be used to assess the sensitivity and suitability of the instruments, estimate the levels of variability in outcomes for future sample size calculations, and determine the willingness of participants to participate in activities, wear fitness trackers, and complete our measures. These results can help to inform a potential future larger-scale study to examine the intervention’s effectiveness. A preliminary economic cost–benefit analysis will also be performed to better understand the potential for this intervention in the workplace for employers.

### Ethics Approval

We have obtained ethics approval from the Monash University Human Research Ethics Committee (Review reference: 2022-30670-71503) for phase 1 of the study. The necessary precautions will be taken to maintain privacy and confidentiality, including using institution-approved secure data storage space, which is password-protected and only accessible by research team members. All participants will provide written consent prior to their participation in activities. Ethics approval for phase 2 will be obtained prior to commencing those study activities.

## Results

This protocol summarizes the initial study plan. This study was funded in June 2021, and ethics approval for Phase 1 was obtained in January 2022. As of August 2022, qualitative interviews with 12 employees have been completed, and the data have been transcribed and analyzed. The findings and more details will be included in the following study results publication.

## Discussion

### Projected Principal Findings

Once completed, this study will further our understanding of the factors that influence healthier behaviors in the workplace, including social influence between employees. It will also result in co-designed, culturally relevant workplace wellness activities with the goal of promoting better health and reducing chronic disease. Finally, the results will also indicate the feasibility of implementing and studying a digital workplace wellness intervention in this setting and can be used to inform a future, larger-scale effectiveness study.

### Comparison to Prior Work

Prior to planning and commencing this study, our team conducted a scoping review of digital workplace wellness interventions in LMICs. This review is under submission, but its main findings include that most research on digital workplace wellness interventions has taken place in high-income countries, and relatively few interventions have been studied in LMICs. Among those studies that have been done in LMICs, digital workplace wellness interventions focus on a range of health issues, including physical activity [22], nutrition [50], smoking cessation [51], and stress [52]. The most common focus is on health behaviors related to chronic disease prevention, as in our study. For example, Ganesan et al [53] studied a digital stepathalon among 26,562 adult employees in India and found evidence of improvements to outcomes such as step count and body weight. Most studies found some evidence of effectiveness and were feasible to implement and acceptable to employees, suggesting that a similar approach in the Malaysian workplace context holds promise in this study.

### Strengths and Limitations

This study has three major strengths. First, this study was designed using 3 theoretical frameworks that describe the individual and social-level factors that contribute to behavior change, and it will leverage the social connections and influence within a workplace to drive healthier behaviors. Based on our recent scoping review (under submission), many previous studies of digital workplace wellness interventions in LMICs either did not use any theory to inform their design or were informed by individual behavior change theories only. Second, this study will employ the co-design methodology to develop the intervention and will contribute to the literature in this area. Though co-design methods are becoming more commonly used in health studies, they are seldom described or evaluated in detail [54]. Further, existing research suggests that the co-design approach is beneficial for researchers, practitioners, research processes, and outcomes [54]. Finally, as mentioned above, most research on digital workplace wellness interventions has taken place in high-income countries. This study will contribute to the evidence from LMICs by studying an intervention in a Malaysian workplace, taking into account the unique multiethnic cultural context of Malaysia, as well as an individual company’s workplace culture.

This study has an important limitation. Phase 2 employs a small sample size and uses nonrandom sampling methods that will not make it possible to test the intervention’s effectiveness. However, the sample size and sampling methods are appropriate for testing feasibility, which is this study’s aim.

## Abbreviations

CAWW: Collective Action for Wellness in the Workplace
CSQ: client satisfaction questionnaire
HR: human resources
HRQoL: health-related quality of life
LMICs: low- and middle-income country
MOS: Medical Outcomes Study
NCDs: non-communicable disease
SCI-2: Sense of Community Index version 2
SCT: social cognitive theory
SF-12v2: Short Form-12 Health Survey Version 2
SF-36: 36-item Short Form Health Survey
SPS-6: 6-item Stanford Presenteeism Scale

## Data Availability

The qualitative data sets generated from interviews and co-design workshops (phase 1) will not be made available, owing to the need to protect data that could lead to the potential identification of employees by their employer. The quantitative, deidentified data sets generated from phase 2 will be made available to other researchers following the study.
